# Energy substrates that fuel fast neuronal network oscillations

**DOI:** 10.3389/fnins.2014.00398

**Published:** 2014-12-05

**Authors:** Lukas V. Galow, Justus Schneider, Andrea Lewen, Thuy-Truc Ta, Ismini E. Papageorgiou, Oliver Kann

**Affiliations:** Institute of Physiology and Pathophysiology and Interdisciplinary Center for Neurosciences (IZN), University of HeidelbergHeidelberg, Germany

**Keywords:** brain energy metabolism, electrophysiology, glycogen phosphorylase, information processing, lactate, mitochondria, monocarboxylate transporter, synaptic transmission

## Abstract

Fast neuronal network oscillations in the gamma-frequency band (30–−100 Hz) provide a fundamental mechanism of complex neuronal information processing in the hippocampus and neocortex of mammals. Gamma oscillations have been implicated in higher brain functions such as sensory perception, motor activity, and memory formation. The oscillations emerge from precise synapse interactions between excitatory principal neurons such as pyramidal cells and inhibitory GABAergic interneurons, and they are associated with high energy expenditure. However, both energy substrates and metabolic pathways that are capable to power cortical gamma oscillations have been less defined. Here, we investigated the energy sources fueling persistent gamma oscillations in the CA3 subfield of organotypic hippocampal slice cultures of the rat. This preparation permits superior oxygen supply as well as fast application of glucose, glycolytic metabolites or drugs such as glycogen phosphorylase inhibitor during extracellular recordings of the local field potential. Our findings are: (i) gamma oscillations persist in the presence of glucose (10 mmol/L) for greater than 60 min in slice cultures while (ii) lowering glucose levels (2.5 mmol/L) significantly reduces the amplitude of the oscillation. (iii) Gamma oscillations are absent at low concentration of lactate (2 mmol/L). (iv) Gamma oscillations persist at high concentration (20 mmol/L) of either lactate or pyruvate, albeit showing significant reductions in the amplitude. (v) The breakdown of glycogen significantly delays the decay of gamma oscillations during glucose deprivation. However, when glucose is present, the turnover of glycogen is not essential to sustain gamma oscillations. Our study shows that fast neuronal network oscillations can be fueled by different energy-rich substrates, with glucose being most effective.

## Introduction

The mammalian brain is a highly oxidative organ owing to the disproportionately large fraction of oxygen consumption compared with the small fraction of the total body mass (in humans about 20% and 2%, respectively) (Rolfe and Brown, 1997; Erecińska and Silver, 2001). This suggests that complex neuronal information processing is associated with high energy expenditure and requires continuous delivery of glucose from the blood (Shulman et al., 2001; Attwell et al., 2010; Kann, 2012). Glucose enters the extracellular space of the brain parenchyma based on a large concentration gradient (5–7 mmol/L in the blood and 1–2 mmol/L in the extracellular space) via glucose transporters (GLUTs) that are located on endothelial cells of the blood-brain-barrier as well as astrocytes (Roberts, 2007; Hertz et al., 2014). For normal conditions, glucose has been considered to be the dominant exogenous energy substrate in the adult brain (Chih and Roberts, 2003; Dienel, 2012). Having a role in different biochemical pathways, glucose metabolism has important functions related to bioenergetics, neurotransmission, and oxidation–reduction (redox) reactions in the brain parenchyma (Bak et al., 2006; Kann and Kovács, 2007; Dienel, 2012). However, neurons are able to utilize exogenous and endogenous energy substrates other than glucose in certain physiological and pathophysiological conditions (Roberts, 2007).

A prominent example is lactate that is generated during neuronal activity through glycogenolysis and/or anaerobic glycolysis (1–2 mmol/L in the extracellular space) or may even enter the brain parenchyma from the blood (Ide et al., 2000; Roberts, 2007; Overgaard et al., 2012; Hertz et al., 2014). Lactate metabolism may also involve complex interactions between neurons and astrocytes (Suzuki et al., 2011; Pellerin and Magistretti, 2012). Many studies on lactate and neuronal activity were made in slice preparations of the hippocampus (Schurr et al., 1988, 1999; Stittsworth and Lanthorn, 1993; Galeffi et al., 2007; Schurr and Payne, 2007; Ivanov et al., 2011, 2014; Hall et al., 2012; Schurr and Gozal, 2012). In all of these studies, neuronal activation was induced by repetitive electrical stimulation, application of neurotransmitters such as glutamate, or Mg^2+^-free recording solution. Notably, either of these experimental tools evokes quite robust neuronal activation, with widely undefined activity states in the local neuronal network, or even spreading (epileptic) depolarization. Thus, detailed information about the capability of different energy substrates to fuel specific, naturally occurring network activity states such as fast neuronal network oscillations in the gamma-frequency band (30–100 Hz) is lacking in the literature.

Gamma oscillations (30–100 Hz) have been observed in many mammalian brain regions, including the hippocampus and the neocortex (Uhlhaas and Singer, 2010). Gamma oscillations reflect synchronous rhythmic fluctuations of the membrane potential in many neurons of a local neuronal network. In the hippocampus, these subthreshold fluctuations are generated by complex and precise synaptic interactions of excitatory pyramidal cells and inhibitory GABAergic interneurons (Whittington and Traub, 2003; Hájos and Paulsen, 2009; Kann, 2012). The synchronizing effect of gamma oscillations permits the coordinated activation of defined sets of neurons that carry and process information (Hájos and Paulsen, 2009; Kann et al., 2014). Therefore, gamma oscillations provide a temporal matrix for complex neuronal information processing during higher brain functions such as sensory perception, motor activity, and memory formation (Paulsen and Moser, 1998; Uhlhaas and Singer, 2010; van Vugt et al., 2010).

Notably, excitatory pyramidal cells and certain subtypes of inhibitory GABAergic interneurons might differ in the energy demands during gamma oscillations and in their capability to utilize energy substrates. Parvalbumin-positive basket cells, for example, generate action potentials at much higher frequency (“fast-spiking”) compared with pyramidal cells during gamma oscillations (30–100 Hz and 1–3 Hz, respectively). Moreover, parvalbumin-positive basket cells synchronize the activity of numerous pyramidal cells by rhythmic inhibition. As prerequisites, theses interneurons have unique electrophysiological properties that are likely associated with extraordinary high energy expenditure (Gulyás et al., 2006; Hu and Jonas, 2014; Kann et al., 2014).

The present study was designed to identify energy substrates that are capable to power gamma oscillation *in vitro*. Gamma oscillations can be reliably induced in hippocampal slices by bath application of low micromolar concentrations of cholinergic receptor agonists such as acetylcholine that mimics input from the septum (Fisahn et al., 1998; Kann et al., 2011). These oscillations share many features with hippocampal gamma oscillations observed *in vivo* (Kann, 2012). We used organotypic hippocampal slice cultures that were maintained on Biopore™ membranes in an interface recording chamber. This experimental approach permits the induction of persistent gamma oscillations, with superior oxygen supply as well as rapid exchange of energy substrates and drugs (Huchzermeyer et al., 2013).

## Materials and methods

### Slice cultures and recording chamber

Animals were purchased from Charles-River (Sulzfeld, Germany) and housed, cared, and killed in accordance with the recommendations of the European Commission and the authorities of Baden-Württemberg (T56/11). Organotypic hippocampal slice cultures were prepared as described (Kann et al., 2003a,b, 2011). In brief, hippocampal slices (400 μm) were cut with a McIlwain tissue chopper (Mickle Laboratory Engineering Company Ltd., Guildford, UK) from 7 to 9 days-old Wistar rats under sterile conditions. Slices were maintained on Biopore™ membranes (Millicell standing inserts, Merck Millipore, Schwalbach, Germany) between culture medium, which consisted of 50% minimal essential medium, 25% Hank's balanced salt solution (Sigma-Aldrich, Taufkirchen, Germany), 25% horse serum (Life Technologies, Darmstadt, Germany), and 2 mM L-glutamine (Life Technologies) at pH 7.3, and humidified normal atmosphere (5% CO_2_, 36.5°C) in an incubator (Heracell, Thermoscientific, Dreieich, Germany). Biopore™ membranes provide high viability and excellent trans-membrane oxygen transport. The culture medium (1 ml) was replaced three times per week. Slice cultures were used after 7–21 days *in vitro* (DIV) (residual thickness of about 200 μm), when the tissue had recovered from the slice preparation and damaged cut surfaces were re-organized (Kann and Kovács, 2007).

For recordings, the intact Biopore™ membrane carrying slice cultures was inserted into the recording chamber. Slice cultures were maintained at the interface between recording solution and ambient gas mixture. Intact Biopore™ membrane inserts ensure constant supply of oxygen and energy substrates from the recording solution (rate 1.8 ml/min) that flows underneath the Biopore™ membrane; the interface condition permits constant oxygen supply from the ambient gas mixture (95% O_2_ and 5% CO_2_, rate 1.5 l/min).

### Recording solutions and drugs

Slice cultures were constantly supplied with pre-warmed (34 ± 1°C) recording solution, i.e., artificial cerebrospinal fluid that contained: 129 mM NaCl, 3 mM KCl, 1.25 mM NaH_2_PO_4_, 1.8 mM MgSO_4_, 1.6 mM CaCl_2_, 26 mM NaHCO_3_, and 10 mM glucose (Sigma-Aldrich). The pH was 7.3 when the recording solution was saturated with the gas mixture (95% O_2_ and 5% CO_2_).

Gamma oscillations were induced by bath application of low concentrations of cholinergic receptor agonist, acetylcholine (2 μmol/L) and acetylcholine-esterase inhibitor, physostigmine (400 nmol/L) (Huchzermeyer et al., 2013). The absence of action potentials (spiking) was induced by bath application of tetrodotoxin, which blocks fast voltage-gated Na^+^-channels. Acetylcholine was from Sigma-Aldrich, physostigmine was from Tocris and tetrodotoxin from Biotrend (Köln, Germany).

For further specific experiments, Na-pyruvate (Sigma-Aldrich), Na-L-lactate (Alfa Aesar, Karlsruhe, Germany), L-glutamine, CP-316819 (5-Chloro-*N*-[(1*S*,2*R*)-2-hydroxy-3-(methoxymethylamino)-3-oxo-1-(phenylmethyl)propyl]-1*H*-in-dole-2-carboxamide; Tocris, R&D Systems GmbH, Wiesbaden-Nordenstadt, Germany) and DAB (1,4-dideoxy-1,4-imino-d-arabinitol; Sigma-Aldrich) were used. Stock solution of DAB was made in double distilled H_2_O and CP-316819 was dissolved in DMSO, with a final solvent fraction of less than 0.001% in the recording solution.

### Recordings of local field potentials

The local field potential (LFP) was recorded with glass electrodes (tip diameter 3–5 μm) that were pulled from GB150F-8P borosilicate filaments (Science Products GmbH, Hofheim, Germany) with a PC-10 vertical micropipette puller (Narishige International Ltd., London, UK) and backfilled with recording solution. The glass electrode was positioned in stratum pyramidale of the CA3 subfield with a mechanical micromanipulator (MM 33, Märzhäuser, Wetzlar). LFPs were recorded with an EXT 10-2F amplifier in EPMS-07 housing (npi electronic GmbH, Tamm, Germany), low-pass filtered at 3 kHz, and digitized at 10 kHz using CED 1401 interface and Spike2 software (Cambridge Electronic Design, Cambridge, UK) for offline analysis.

### Toluidine blue staining

Slice cultures were fixed in paraformaldehyde (4%, 0.1 M phosphate buffer; Applichem, Darmstadt, Germany) and rinsed in 0.1 M phosphate-buffered salt solution (PBS). Thereafter, slice cultures were exposed for 20 min to toluidine-blue working solution, which was a mixture of 5 ml stock solution (1 g of Toluidine Blue O in 100 ml of 70% ethanol; Sigma-Aldrich) and 45 ml of 1% NaCl solution (pH 2.0–2.5). Thereafter, 96% ethanol (100 ml of 96% ethanol and 4 drops of acetic acid) was used for color-differentiation of the staining. The differentiation step with strong acid removes unspecific staining of weak acidic structures and, thus, increases the contrast between background and stained cells. The process was stopped using 0.1 M PBS, once the differentiation was clearly visible. After brief rinsing with double distilled water, slice cultures were placed on object plates and dried overnight. The slices were then exposed to xylol (Sigma-Aldrich) for 10 min and embedded with Entellan Neu (Merck Millipore, Schwalbach, Germany).

### Data analysis

Offline signal analysis of gamma oscillations was performed in MatLab 11.0 (MathWorks). Data segments of 100 s were low-pass filtered with a digital Butterworth algorithm at 200 Hz corner frequency and processed with Welch's algorithm and Fast Fourier Transformation with a Hamming window size of 4096 points for calculation of the power spectral density and the power spectrum, respectively (bin size = 2.441 Hz). Gamma oscillations were analyzed for various parameters, i.e., peak frequency (Freq), area under curve (AUC), full width at half maximum (FWHM), peak power spectral density (PSD), amplitude (Ampl), and variance of the amplitude (Var).

Data are presented as mean ± SD derived from (n) slice cultures and (N) preparations of rat pups. Statistical significance (*P* < 0.05) was determined using SigmaPlot 12.5 (Systat Software, Inc., San Jose, CA, USA). Data distribution was tested for normality with Shapiro-Wilk test. Comparisons between paired data were made with paired *t*-test or Wilcoxon signed rank test. For multiple variance comparison, One-Way ANOVA or Kruskal-Wallis One-Way ANOVA on ranks with Dunn's *post-hoc* test was used for unpaired data and One-Way repeated measures ANOVA with Holm-Sidak *post-hoc* test or Friedman repeated measures ANOVA on ranks with Tukey *post-hoc* test was used for paired data. Figures were generated using Excel (Microsoft Corporation, Redmond, USA) and CorelDRAW (Corel Corporation, Ottawa, Ontario, Canada).

## Results

### Gamma oscillations in the presence of glucose

**Figure 1:**
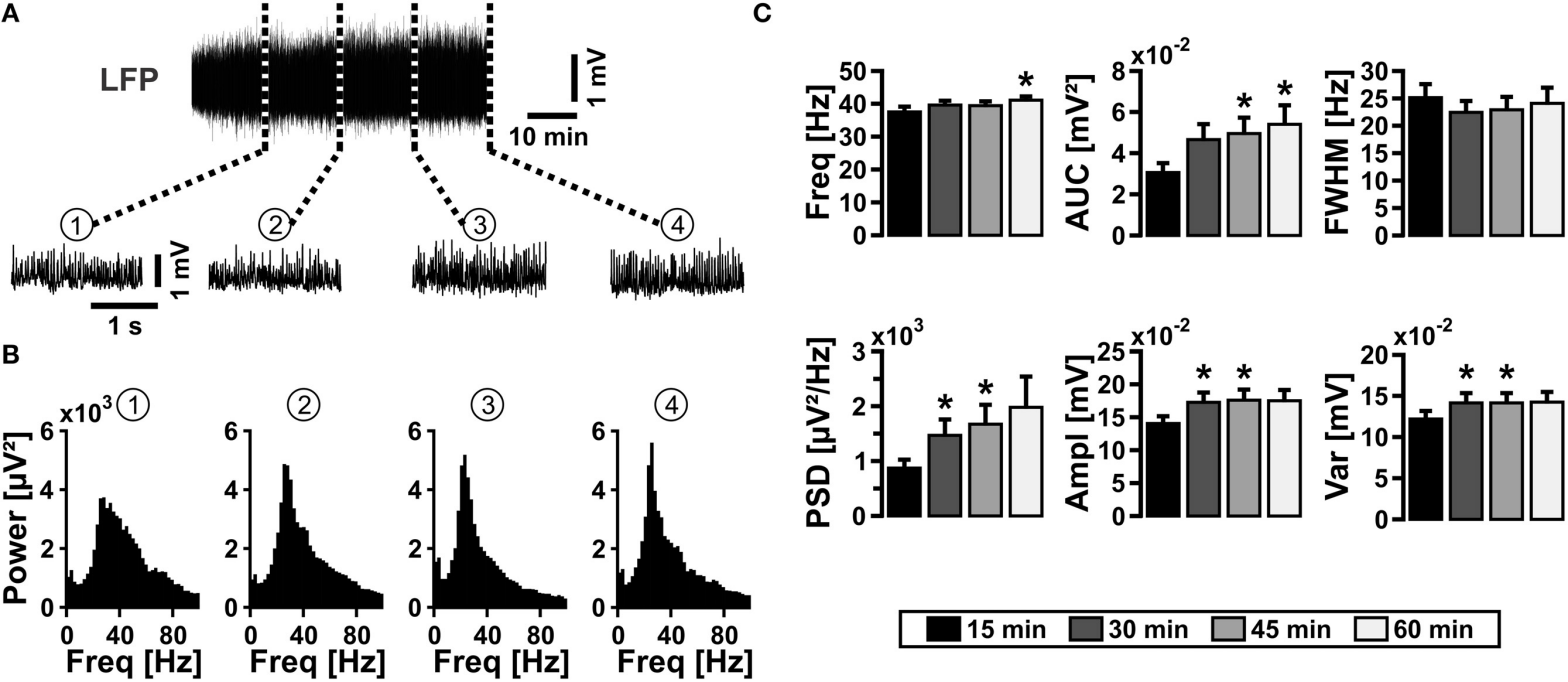
**Gamma oscillations in slice cultures**. **(A)** Gamma oscillations were induced by bath application of acetylcholine (2 μmol/L) and physostigmine (400 nmol/L) (upper sample trace), and they persisted for more than 60 min (*n* = 13, *N* = 6). Gamma oscillations are shown with a higher temporal resolution after 15 min (1), 30 min (2), 45 min (3), and 60 min (4) (lower sample traces). Local field potentials (LFP) were recorded in stratum pyramidale of the CA3 subfield in organotypic hippocampal slice cultures of the rat. **(B)** Corresponding power spectra of sample traces shown in **(A)** were calculated from 100 s taken at the end of each data segment. **(C)** Gamma oscillations were analyzed for various parameters, i.e., peak frequency (Freq), area under curve (AUC), full width at half maximum (FWHM), peak power spectral density (PSD), amplitude (Ampl), and variance of the amplitude (Var). Friedman repeated-measures ANOVA on ranks and Tukey *post-hoc* test. Statistical significance is marked by asterisks (*P* < 0.05).

We induced gamma oscillations by bath application of acetylcholine in organotypic hippocampal slice cultures and performed extracellular recordings of the local field potential (LFP) in stratum pyramidale of the CA3 subfield (Kann et al., 2011). In standard recording solution, i.e., in the presence of glucose (10 mmol/L) and high oxygen fraction (95%) in the ambient atmosphere (Kann and Kovács, 2007), gamma oscillations were fully established after about 15 min of acetylcholine application (Figures 1A–C). The oscillations were characterized by a frequency of around 40 Hz in the power spectrum and persisted for 60 min (Figure 1C) and longer (data not shown). This experiment demonstrates that the reduced composition of recording solution as well as the supply of oxygen in excess for 1 h does not result in evident functional disturbances in the local neuronal network of the CA3 subfield.

**Figure 2:**
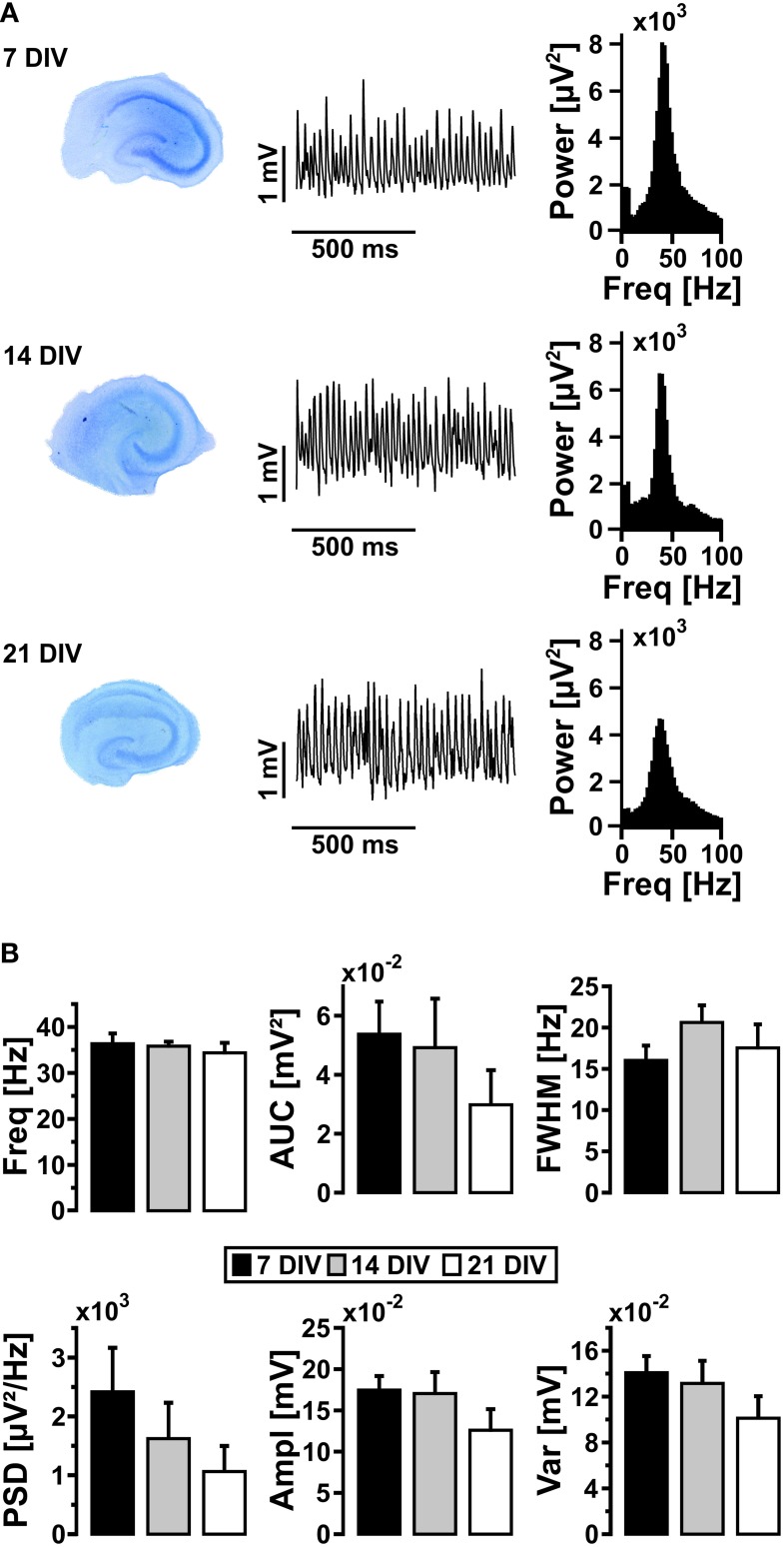
**Gamma oscillations and maturation of slice cultures**. **(A)** Slice cultures were stained with toluidine blue after 7, 14, and 21 DIV (left). Gamma oscillations were induced by bath application of acetylcholine (2 μmol/L) and physostigmine (400 nmol/L) and local field potentials (LFP) were recorded in stratum pyramidale of the CA3 subfield (middle). Corresponding power spectra of the sample traces were calculated from data segments of 100 s (right). **(B)** Gamma oscillations were analyzed for various parameters, i.e., peak frequency (Freq), area under curve (AUC), full width at half maximum (FWHM), peak power spectral density (PSD), amplitude (Ampl) and variance of the amplitude (Var) (7 DIV, *n* = 12, *N* = 4; 14 DIV, *n* = 7, *N* = 3; 21 DIV, *n* = 6, *N* = 3). One-Way ANOVA and Kruskal-Wallis ANOVA on ranks.

We next tested whether the properties of persistent gamma oscillations changed with maturation of slice cultures (Bahr et al., 1995; De Simoni et al., 2003). The characteristics of gamma oscillations in standard recording condition (10 mmol/L glucose, 95% oxygen fraction) did not significantly change after 21 DIV (Figure 2), albeit the well-known decrease of slice thickness over time in culture (Bahr et al., 1995; Kann and Kovács, 2007). For further experiments, we used slice cultures after 7 DIV and up to 14 DIV.

Bath application of tetrodotoxin (1 μmol/l), which blocks fast voltage-gated Na^+^-channels and thus action potentials, suppressed gamma oscillations after about 5 min (data not shown). This experiment reveals that maintenance of slice cultures on Biopore™ membranes in the interface recording chamber permits rapid drug application and tissue saturation.

**Figure 3:**
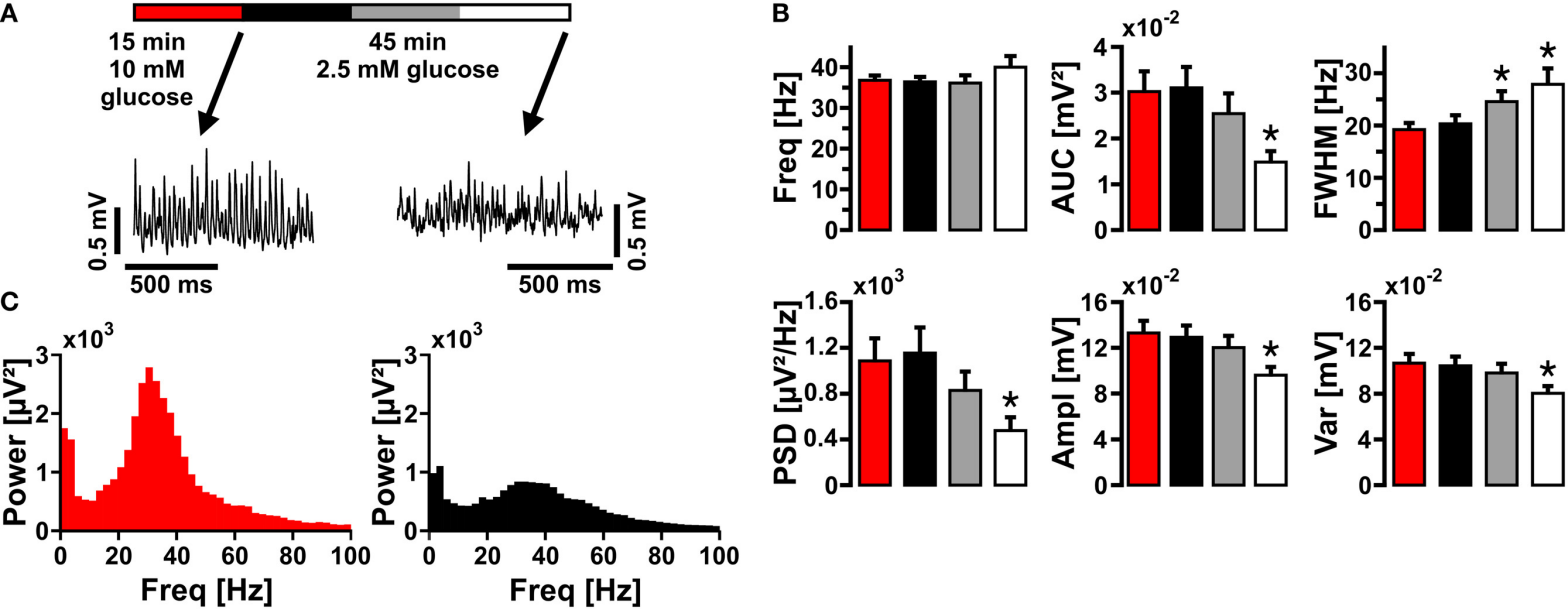
**Gamma oscillations at low glucose concentration**. **(A)** Gamma oscillations were induced by bath application of acetylcholine (2 μmol/L) and physostigmine (400 nmol/L) in the presence of 10 mmol/L glucose (red). Then, glucose was lowered to 2.5 mmol/L in the recording solution and the properties of gamma oscillations were analyzed after 15 min (black), 30 min (gray), and 45 min (white). Local field potentials (LFP) were recorded in stratum pyramidale of the CA3 subfield (sample traces). **(B)** Corresponding power spectra of sample traces shown in **(A)** were calculated from 100 s taken at the end of each data segment. **(C)** Gamma oscillations were analyzed for various parameters, i.e., peak frequency (Freq), area under curve (AUC), full width at half maximum (FWHM), peak power spectral density (PSD), amplitude (Ampl), and variance of the amplitude (Var) (*n* = 14, *N* = 3). Note the decrease in amplitude at low glucose concentration. Repeated-measures ANOVA and Holm-Sidak *post-hoc* test or Friedman repeated-measures ANOVA on ranks and Tukey *post-hoc* test. Statistical significance is marked by asterisks (*P* < 0.05).

We further addressed whether application of glucose in a concentration closer to physiological conditions (2.5 mmol/L) would affect gamma oscillations (Roberts, 2007; Schurr and Payne, 2007). In this condition, gamma oscillations were still present. However, there were significant disturbances in the characteristics of gamma oscillations such as lower amplitude (AUC, PSD, Ampl) and widening of FWHM, reflecting less numbers and less synchrony of activated synapses, respectively (Figure 3). This experiment shows that even in optimized recording condition, i.e., utilization of Biopore™ membranes and interface recording chamber, a larger concentration gradient of glucose is required to fuel gamma oscillations *in vitro*.

### Lactate and pyruvate as energy substrates

**Figure 4:**
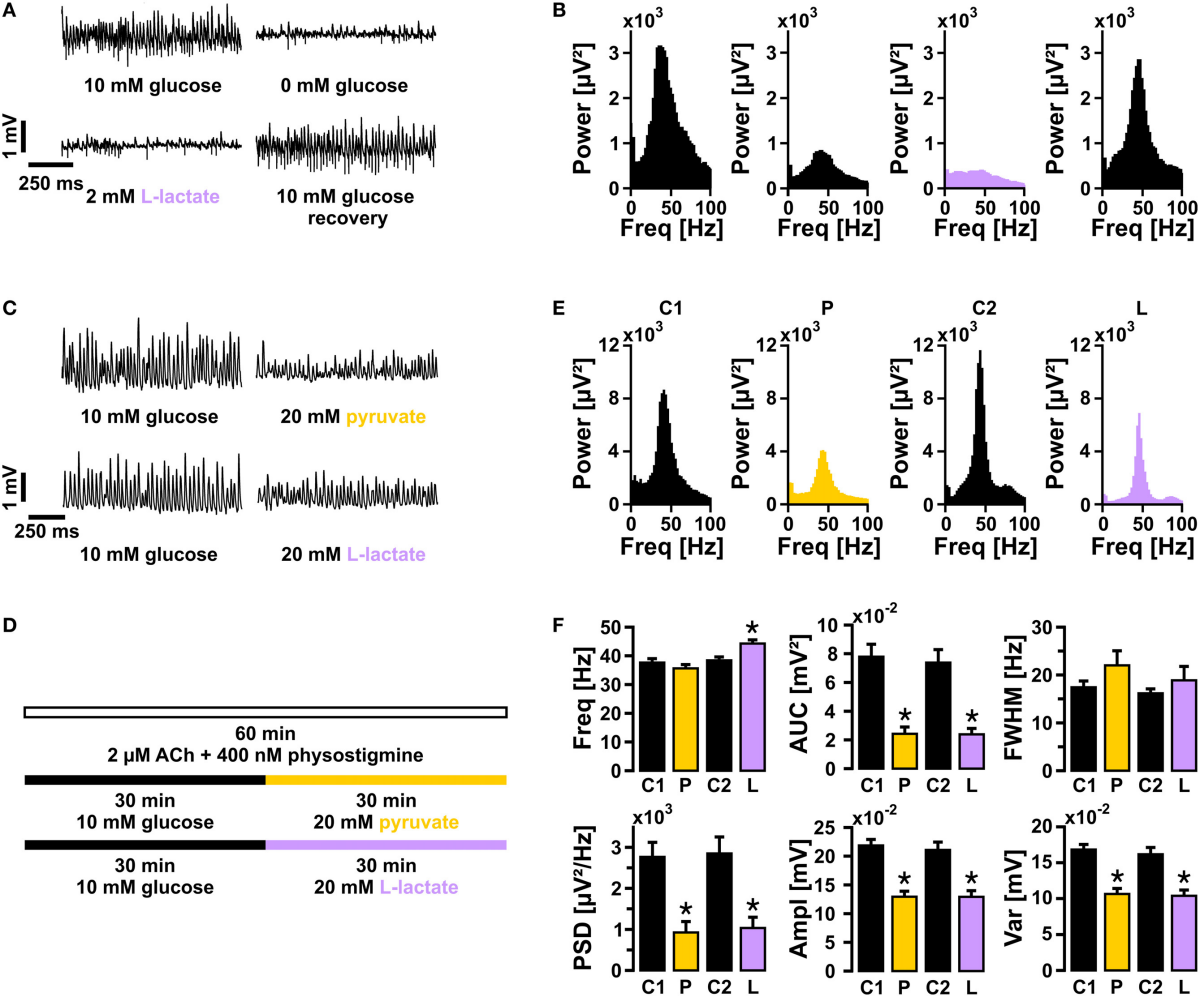
**Gamma oscillations in the presence of either lactate or pyruvate. (A)** Gamma oscillations were induced by bath application of acetylcholine (2 μmol/L) and physostigmine (400 nmol/L). Initially, the recording solution contained 10 mmol/L glucose. Subsequently, recording solutions containing 0 mmol/L glucose (15 min), 2 mmol/L lactate (20 min), and again 10 mmol/L glucose were applied (*n* = 15, *N* = 3), while local field potentials (LFP) were recorded in stratum pyramidale of the CA3 subfield (sample traces). **(B)** Corresponding power spectra of sample traces shown in **(A)** were calculated from 100 s taken at the end of each data segment. **(C)** Sample traces of gamma oscillations in the presence of 10 mmol/L glucose vs. 20 mmol/L pyruvate (upper traces) or 20 mmol/L lactate (lower traces), according to the protocol given in **(D)**. **(E)** Corresponding power spectra of sample traces shown in **(C)** were calculated from data segments of 100 s. **(F)** Gamma oscillations were analyzed for various parameters, i.e., peak frequency (Freq), area under curve (AUC), full width at half maximum (FWHM), peak power spectral density (PSD), amplitude (Ampl), and variance of the amplitude (Var) (control 1 and pyruvate, *n* = 12, *N* = 4; control 2, and lactate, *n* = 13, *N* = 4). Note the significant decrease in amplitude even at the high concentration of lactate or pyruvate. Paired *t*-test or Wilcoxon signed rank test. Statistical significance is marked by asterisks (*P* < 0.05).

We next tested whether lactate in a concentration of 2 mmol/L was capable to fuel gamma oscillations, similar as reported for neuronal population responses evoked by electrical stimulation (Schurr et al., 1988). To exclude that breakdown of the glycogen reserve in slice cultures (Cater et al., 2001) affected the outcome of this experiment (see below), we first depleted the glycogen stores by glucose deprivation (recording solution with 0 mmol/L glucose) for 15 min, which widely resulted in suppression of gamma oscillations (Figures 4A–B). Subsequent bath application of lactate (2 mmol/L) for 20 min did not rescue gamma oscillations. By contrast, re-application of glucose (10 mmol/L) resulted in almost full recovery of the oscillations.

Subsequently, we determined the capability of equicaloric concentrations of lactate (20 mmol/L) or pyruvate (20 mmol/L) to fuel gamma oscillations (Cater et al., 2003; Galeffi et al., 2007; Gandhi et al., 2009). Either of these substrates could indeed sustain gamma oscillations for 30 min (Figures 4C–F) and longer (data not shown). However, in the presence of lactate or pyruvate gamma oscillations showed significantly lower amplitudes (AUC, PSD, Ampl) compared with controls (10 mmol/L glucose); lactate significantly increased the frequency of the oscillations.

**Figure 5:**
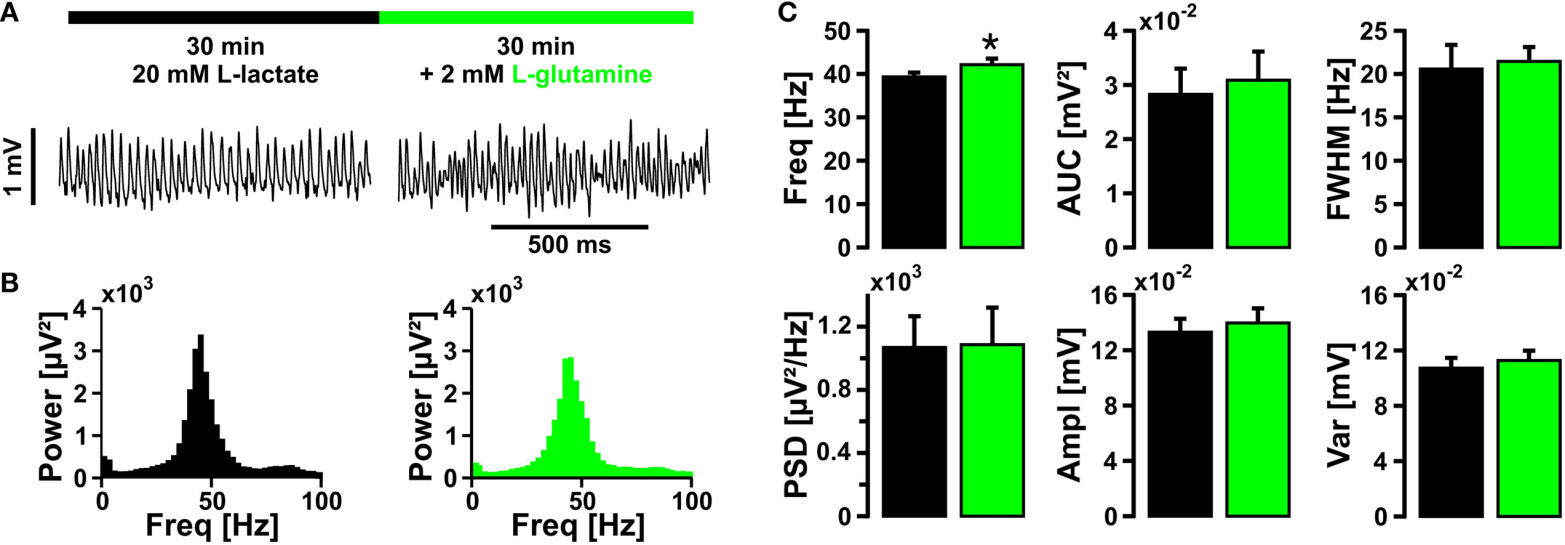
**Gamma oscillations in the presence of lactate and glutamine**. **(A)** Gamma oscillations were induced by bath application of acetylcholine (2 μmol/L) and physostigmine (400 nmol/L) in the presence of 20 mmol/L lactate (black bar); after 30 min, 2 mmol/L glutamine was added (green bar). Local field potentials (LFP) were recorded in stratum pyramidale of the CA3 subfield (sample traces). **(B)** Corresponding power spectra of sample traces shown in **(A)** were calculated from 100 s taken at the end of each data segment. **(C)** Gamma oscillations were analyzed for various parameters, i.e., peak frequency (Freq), area under curve (AUC), full width at half maximum (FWHM), peak power spectral density (PSD), amplitude (Ampl), and variance of the amplitude (Var) (*n* = 19, *N* = 4). Note that glutamine has only a minor effect on the frequency of gamma oscillations. Paired *t*-test. Statistical significance is marked by asterisks (*P* < 0.05).

Supporting lactate (20 mmol/L) with glutamine (2 mmol/L), which is an important precursor for neurotransmitters, glutamate, and GABA (Waagepetersen et al., 1998; Hertz et al., 2014), did not rescue the amplitude of gamma oscillations (Figure 5).

These experiments show that a high concentration of either lactate or pyruvate can basically power gamma oscillations but alters their characteristics.

### Glycogen stores and inhibition of glycogen phosphorylase

**Figure 6:**
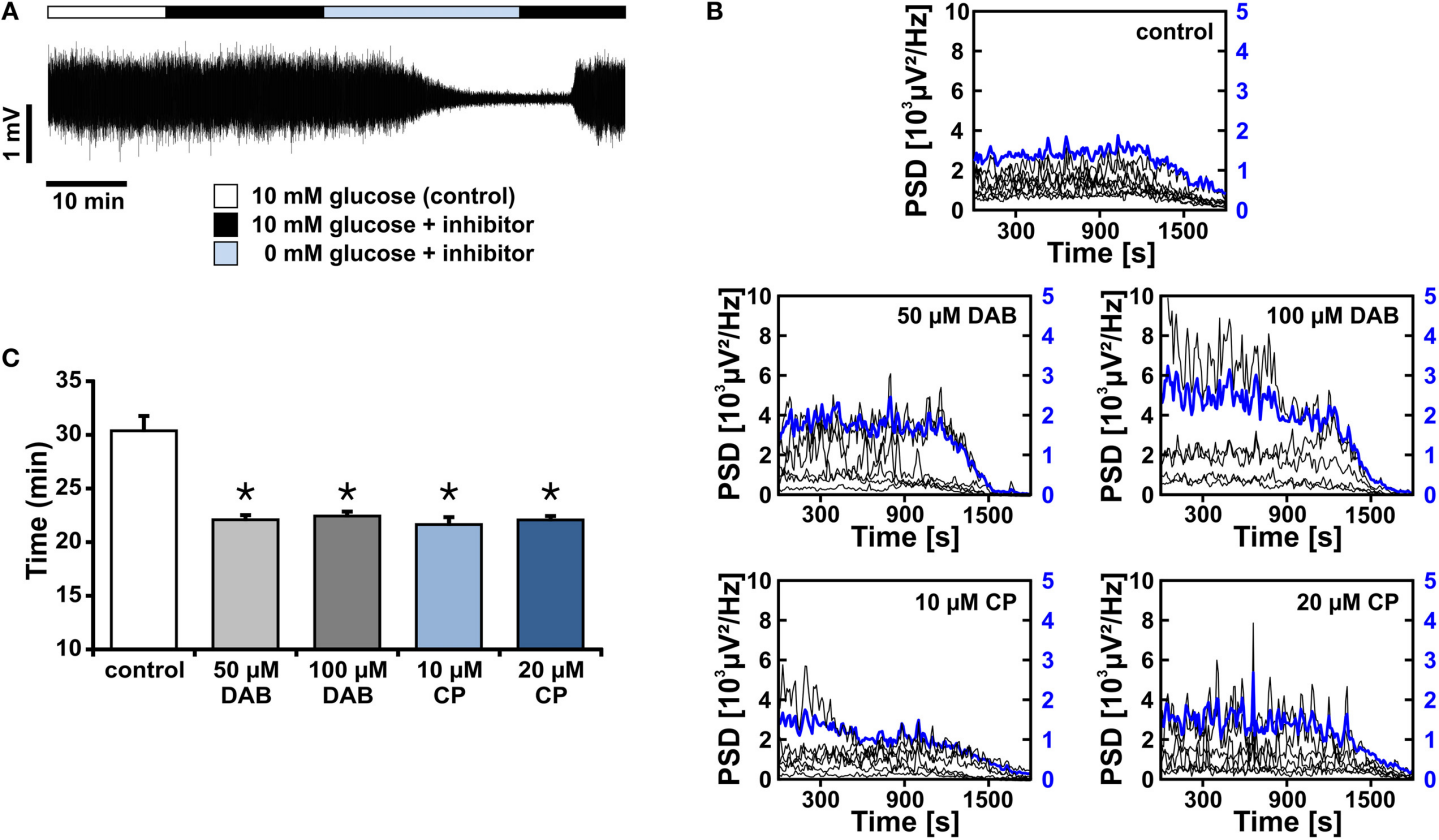
**Gamma oscillations and glycogen stores during glucose deprivation**. **(A)** Gamma oscillations were induced by bath application of acetylcholine (2 μmol/L) and physostigmine (400 nmol/L) in the presence of 10 mmol/L glucose (white bar). Subsequently, inhibitors of glycogen phosphorylase, DAB (50 μmol/l or 100 μmol/L) or CP-316819 (10 μmol/L or 20 μmol/L) were applied, in the presence (black bar) or absence (light blue bar) of glucose. Note that the standard gas mixture (95% O2 and 5% CO2) was continuously present. Local field potentials (LFP) were recorded in stratum pyramidale of the CA3 subfield subfield (sample trace). **(B)** The peak power spectral density (μV^2^/Hz) for each recording trace is shown in black (scaling on left y-axis), the average of all recordings is shown in blue (scaling on right y-axis). Power spectra were calculated every 10 s and plotted over time. **(C)** The points in time are given for complete suppression of gamma oscillations, i.e., power reaching a threshold defined as the mean of the last 100 s plus 1 standard deviation, according to the protocol given in **(A)** (control, *n* = 10, *N* = 3; DAB, *n* = 6, *N* = 3, and *n* = 5, *N* = 3; CP-316819, *n* = 6, *N* = 3, and *n* = 5, *N* = 2). Note that inhibition of glycogen phosphorylase accelerates the decay of gamma oscillations during glucose deprivation. Kruskal Wallis ANOVA on ranks. Statistical significance vs. control is marked by asterisks (*P* < 0.05).

We further explored whether glycogen breakdown is capable to fuel gamma oscillations. Glycogen stores have been described in astrocytes and, more recently, also in neurons (Choi et al., 2012; Dienel and Cruz, 2014; Saez et al., 2014). At first, we determined the time course of suppression of gamma oscillations during glucose deprivation in the presence of 95% oxygen fraction (Figures 6A–C). Activity was completely suppressed after 29 ± 1 min (*n* = 10) in recording solution with 0 mmol/L glucose (data not shown). This time course reflects utilization of various energy reserves such as glycogen for ATP generation in different pathways (Roberts, 2007; Dienel and Cruz, 2014) and, presumably, a considerable glycogen reserve in slice cultures (Cater et al., 2001).

We next pharmacologically blocked glycogen phosphorylase, which is a crucial enzyme for glycogen breakdown and thus serves in the initiation of glycogen metabolism (Dienel and Cruz, 2014). We applied two different inhibitors, i.e., DAB and CP-316819, at various concentrations (Gibbs et al., 2006; Dienel et al., 2007; Suh et al., 2007; Walls et al., 2008; Sickmann et al., 2009). Blockade of glycogen phosphorylase resulted in significantly faster suppression of gamma oscillations of 8 ± 1 min (*n* > 10) during glucose deprivation, indicating that glycogen breakdown can indeed support the maintenance of fast neuronal-network oscillations and thus higher brain functions in situations when glucose supply becomes limited (Wender et al., 2000; Abdelmalik et al., 2007).

**Figure 7:**
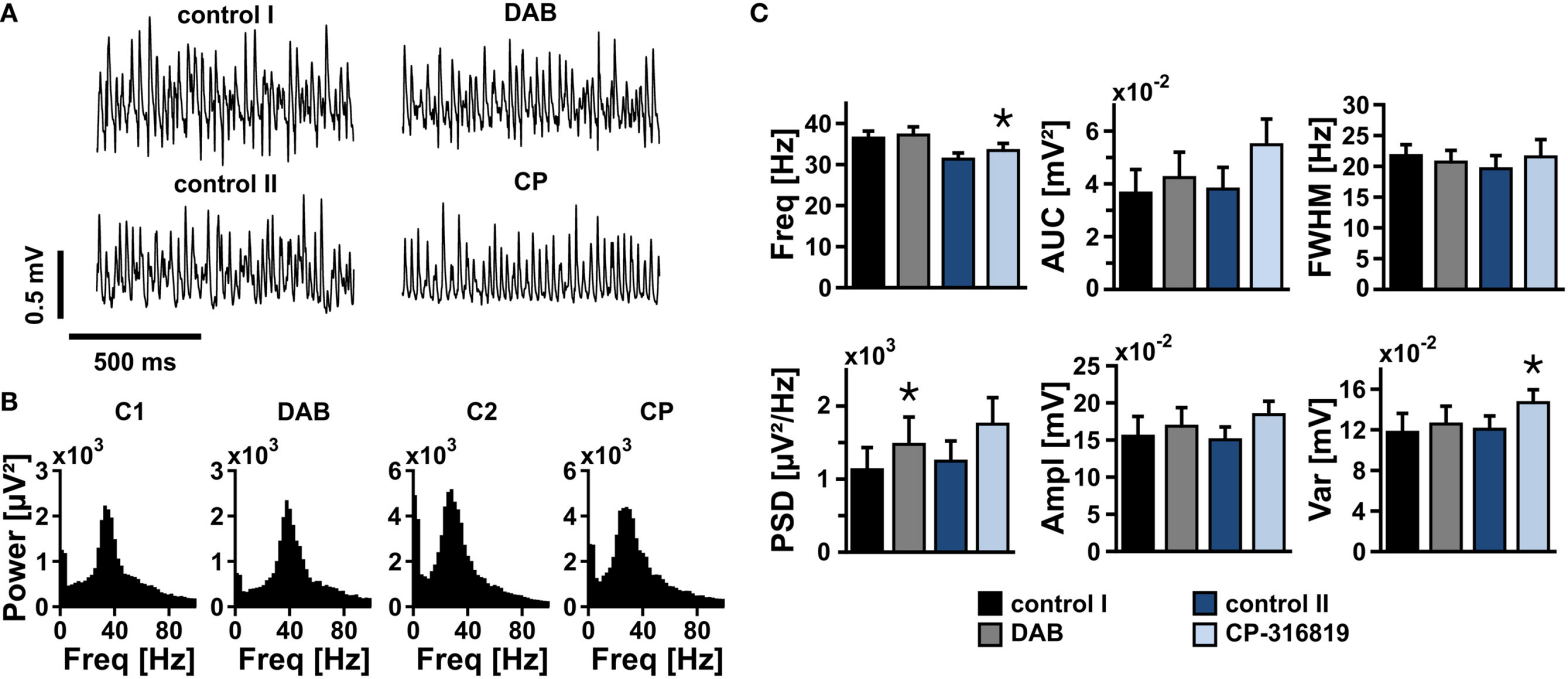
**Gamma oscillations and glycogen turnover**. **(A)** Gamma oscillations were induced by bath application of acetylcholine (2 μmol/L) and physostigmine (400 nmol/L) in the presence of 10 mmol/L glucose. After 15 min, inhibitors of glycogen phosphorylase, DAB (50 μmol/l or 100 μmol/L) or CP-316819 (10 μmol/L or 20 μmol/L) were added to the recording solution. Local field potentials (LFP) were recorded in stratum pyramidale of the CA3 subfield (sample traces). **(B)** Corresponding power spectra of sample traces shown in **(A)** were calculated from 100 s taken at the end of each data segment. **(C)** Gamma oscillations were analyzed for various parameters, i.e., peak frequency (Freq), area under curve (AUC), full width at half maximum (FWHM), peak power spectral density (PSD), amplitude (Ampl), and variance of the amplitude (Var) (control 1 and DAB, *n* = 13, *N* = 4; control 2 and CP-316819, *n* = 10, *N* = 3). Note that inhibition of glycogen phosphorylase in the presence of 10 mmol/L glucose has only minor effects on gamma oscillations. Paired *t*-test or Wilcoxon signed rank test. Statistical significance is marked by asterisks (*P* < 0.05).

We finally tested whether the turnover of glycogen was essential for sustainment of gamma oscillations. After gamma oscillations had been fully established, bath application of DAB (100 μmol/L) or CP-316819 (20 μmol/L) for 20 min left gamma oscillations widely intact (Figures 7A–C). We even partially observed increases in power and frequency of the oscillations.

These data show that glycogen is an important fuel reserve to sustain gamma oscillations for a transient period under pathological conditions such as hypoglycemia. However, the turnover of glycogen does not significantly contribute to sustainment of gamma oscillations when glucose is present.

## Discussion

### Gamma oscillations in organotypic hippocampal slice cultures

Gamma oscillations provide a temporal matrix for complex neuronal information processing during higher brain functions such as sensory perception, motor activity, and memory formation (Paulsen and Moser, 1998; Uhlhaas and Singer, 2010; Kann et al., 2014).

Persistent gamma oscillations can be reliably induced in acute slices and slice cultures of the hippocampus by bath application of cholinergic receptor agonists such as acetylcholine, which mimics cholinergic neuronal input from the septum *in vivo* (Bartos et al., 2007). Acetylcholine activates primarily muscarinic receptors in pyramidal cells and interneurons that interact via complex synaptic mechanisms (Bartos et al., 2007; Hájos and Paulsen, 2009). Cholinergic receptor activation finally leads to subthreshold membrane potential fluctuations in neurons and concomitant rhythmic network oscillations as detected by extracellular recordings of the LFP (Hájos et al., 1998; Fisahn et al., 2002; Kann et al., 2014). Notably, fast rhythmic GABAergic inhibition by parvalbumin-positive fast-spiking interneurons is crucial for the generation of gamma oscillations (Bartos et al., 2007; Gulyás et al., 2010; Oren et al., 2010). Cholinergically induced hippocampal gamma oscillations *in vitro* are most prominent in the CA3 subfield (Fisahn et al., 1998; Kann et al., 2011), which is similar to the pattern of gamma oscillations *in vivo* (Penttonen et al., 1998; Montgomery and Buzsáki, 2007). Gamma oscillations *in vivo* occur transiently on the 100 ms time scale upon sensory input or during specific cognitive tasks. In the human brain, for example, gamma oscillations can last in the range of minutes, dependent on the task (Lehmann et al., 2001; Lutz et al., 2004). This aspect is important for the validation of *in vitro* models, most of which feature persistent gamma oscillations (Bartos et al., 2007; Hájos and Paulsen, 2009).

Recent *in vitro* and *in vivo* studies demonstrated that gamma oscillations were associated with high energy expenditure (Niessing et al., 2005; Nishida et al., 2008; Kann et al., 2011; Huchzermeyer et al., 2013), which is most likely caused by increased rates of action potentials and synaptic interactions. In particular, the significant increase in excitatory and inhibitory postsynaptic potentials during gamma oscillations elicits strong ion fluxes across the neuronal membrane of pyramidal cells and inhibitory interneurons that finally need to be restored by ATP-dependent ion pumps such as Na^+^/K^+^-ATPase (Kann et al., 2014).

Taken together, persistent gamma oscillations in organotypic hippocampal slice cultures are a useful model for a specific neuronal network activity state with high energy expenditure that naturally occurs *in vivo*.

### Glucose, lactate and pyruvate as energy substrates

Here we show that gamma oscillations reliably persist for more than 1 h in standard recording condition, i.e., in the presence of 10 mmol/L glucose and 95% oxygen fraction. Traditionally, brain slices are maintained in the presence of 10 mmol/L glucose for two main reasons: (i) improved recovery from the preparation procedure and (ii) heterogeneity in glucose and oxygen availability owing to the use of interface or submerged conditions, which also includes different application rates of recording solution (Li and McIlwain, 1957; Kann and Kovács, 2007). Our optimized recording condition, i.e., the combination of slice cultures, Biopore™ membranes, and interface recording chamber, features superior supply of energy substrates and oxygen as well as fast drug application (Huchzermeyer et al., 2013).

However, gamma oscillations showed clear disturbances in the presence of 2.5 mmol/L glucose, even in the optimized recording condition. This finding differs from experiments in acute hippocampal slices, in which 2.5 mmol/L glucose were sufficient to sustain neuronal population responses as evoked by electrical stimulation (Schurr and Payne, 2007). Our experiments with energy substrates other than glucose showed that a high concentration (20 mmol/L) of lactate or pyruvate could indeed sustain gamma oscillations, but these oscillations were of significantly lower amplitude. This is also in contrast with other studies showing that lactate at 2 mmol/L or higher could maintain synaptic function in hippocampal tissue *in vitro* as well as, or better than, glucose (Schurr et al., 1988; Schurr and Payne, 2007; Ivanov et al., 2011) and/or proposing that lactate is the preferred energy substrate of neurons (Bouzier-Sore et al., 2003).

These different findings on the capability of low glucose or lactate to sustain neuronal activity are most likely due to (i) the respective activity state that is induced in the local neuronal network and/or (ii) intracellular acidification of neurons in the presence of lactate or pyruvate.

- In previous studies, electrical stimulation with either single (Schurr et al., 1988; Schurr and Payne, 2007) or repetitive pulses (at 10 Hz, for 10 or 30 s) (Ivanov et al., 2011) was used in acute hippocampal slices. Such electrical stimulation is quite robust and brief, and it enforces all neurons in the local network to generate action potentials. The final individual action potential frequency may considerably vary depending on neuronal subtypes and accommodation characteristics (Kann et al., 2014). By contrast, hippocampal gamma oscillations are a specific, naturally occurring network activity state that is based on complex and precise synaptic interactions between excitatory pyramidal cells and inhibitory GABAergic interneurons (Whittington and Traub, 2003; Bartos et al., 2007; Hájos and Paulsen, 2009). During gamma oscillations, fast-spiking interneurons generate action potentials at 30–100 Hz while pyramidal cells spike at 1–3 Hz *in vitro* and *in vivo* (Csicsvari et al., 1999; Traub et al., 2000; Hájos et al., 2004; Gloveli et al., 2005). In particular, parvalbumin-positive fast-spiking interneurons are crucial for the generation of gamma oscillations and have very special characteristics such as formation of a basket cell network (Ribak et al., 1993; Traub et al., 2001), perisomatic control of pyramidal cells (Hájos et al., 2004; Gloveli et al., 2005), rapid action potential kinetics and high sodium entry ratio (Carter and Bean, 2009; Hu and Jonas, 2014). Moreover, parvalbumin-positive fast-spiking interneurons contain large numbers of mitochondria (Kageyama and Wong-Riley, 1982; Gulyás et al., 2006; Takács et al., 2014), which likely reflects the extraordinary high energy expenditure of this neuronal subtype during fast network oscillations (Kann et al., 2014). In agreement with these biophysical and biochemical characteristics, fast-spiking interneurons and gamma oscillations are exquisitely sensitive to metabolic stress (Huchzermeyer et al., 2008; Kann et al., 2011; Whittaker et al., 2011). The inability of 2 mmol/L glucose and 20 mmol/L lactate or pyruvate to (fully) sustain gamma oscillations might thus be related to properties of fast-spiking interneurons such as high energy expenditure, limitations in the uptake of substrates via glucose transporter GLUT-3 and monocarboxylate transporter MCT-2 and/or limitations by rate-limiting enzymes in cytoplasmic and mitochondrial pathways related to energy and neurotransmitter metabolism (Waagepetersen et al., 1998; Bak et al., 2006; Simpson et al., 2007; Barros, 2013). However, the properties of transporters and enzymes in parvalbumin-positive fast-spiking interneurons are widely unknown (Kann et al., 2014).
- A complementary or alternative explanation for the disturbances in gamma oscillations might be intracellular neuronal acidification in the presence of high concentration of lactate or pyruvate. Both substrates are taken up by neurons via MCT-2, which is a proton-linked monocarboxylate transporter (Roberts, 2007; Halestrap, 2013). Previous studies indeed showed decreases in the intracellular pH in the presence of 20 mmol/L lactate (Munsch and Pape, 1999; Ruusuvuori et al., 2010).

### Glycogen stores and inhibition of glycogen phosphorylase

Glycogen phosphorylase catalyzes the rate-limiting step in glycogenolysis in animals by releasing glucose-1-phosphate from the terminal alpha-1,4-glycosidic bond, finally supporting various intracellular metabolic pathways (Dienel and Cruz, 2014). It is widely accepted that glycogen is stored in astrocytes (Roberts, 2007; Dienel and Cruz, 2014). However, glycogen has been also found in localized compartments of neurons such as synaptic boutons and dendritic spines (Fiala et al., 2003), and there is recent evidence that neurons have an active glycogen metabolism (Saez et al., 2014). Several studies support two roles of glycogen as an energy substrate. The first role is to supply energy for regular neuronal activity (Swanson, 1992; Dienel et al., 2002; Kong et al., 2002; Gibbs et al., 2006; Choi et al., 2012; Duran et al., 2013). The second role is to provide glucose equivalents when supply with glucose or oxygen is limited, such as during hypoglycemia or ischemia/hypoxia (Wender et al., 2000; Choi et al., 2003; Saez et al., 2014). The glycogen metabolism might primarily occur in astrocytes that finally provide lactate to neurons (Dringen et al., 1993; Dienel and Cruz, 2014).

In our study, glycogen breakdown significantly delayed the decay of gamma oscillations in the absence of glucose for about 8 min. This indicates (i) a considerable glycogen reserve in slice cultures that might be larger than *in vivo* (Cater et al., 2001; Kong et al., 2002), and (ii) quite effective mechanisms to mobilize and metabolize astrocytic and neuronal glycogen during gamma oscillations. However, inhibition of glycogen phosphorylase had only minor effects on gamma oscillations in the presence of glucose. Thus, our data suggest that glycogen serves as an important fuel reserve to sustain gamma oscillations and thus higher brain functions for a transient period under pathological conditions.

### Conflict of interest statement

The authors declare that the research was conducted in the absence of any commercial or financial relationships that could be construed as a potential conflict of interest.

## Abbreviations

Ampl: amplitude
AUC: area under curve
DIV: days *in vitro*
Freq: peak frequency
FWHM: full width at half maximum
LFP: local field potential
PSD: peak power spectral density
Var: variance of the amplitude.
